# Improving the accuracy of estimates of animal path and travel distance using GPS drift‐corrected dead reckoning

**DOI:** 10.1002/ece3.2359

**Published:** 2016-08-03

**Authors:** Oliver P. Dewhirst, Hannah K. Evans, Kyle Roskilly, Richard J. Harvey, Tatjana Y. Hubel, Alan M. Wilson

**Affiliations:** ^1^ Structure & Motion LaboratoryThe Royal Veterinary College, University of LondonHatfieldAL9 7TAUK

**Keywords:** Animal energetics, animal locomotion, animal travel distance, dead reckoning, GPS tracking collar

## Abstract

Route taken and distance travelled are important parameters for studies of animal locomotion. They are often measured using a collar equipped with GPS. Collar weight restrictions limit battery size, which leads to a compromise between collar operating life and GPS fix rate. In studies that rely on linear interpolation between intermittent GPS fixes, path tortuosity will often lead to inaccurate path and distance travelled estimates. Here, we investigate whether GPS‐corrected dead reckoning can improve the accuracy of localization and distance travelled estimates while maximizing collar operating life. Custom‐built tracking collars were deployed on nine freely exercising domestic dogs to collect high fix rate GPS data. Simulations were carried out to measure the extent to which combining accelerometer‐based speed and magnetometer heading estimates (dead reckoning) with low fix rate GPS drift correction could improve the accuracy of path and distance travelled estimates. In our study, median 2‐dimensional root‐mean‐squared (2D‐RMS) position error was between 158 and 463 m (median path length 16.43 km) and distance travelled was underestimated by between 30% and 64% when a GPS position fix was taken every 5 min. Dead reckoning with GPS drift correction (1 GPS fix every 5 min) reduced 2D‐RMS position error to between 15 and 38 m and distance travelled to between an underestimation of 2% and an overestimation of 5%. Achieving this accuracy from GPS alone would require approximately 12 fixes every minute and result in a battery life of approximately 11 days; dead reckoning reduces the number of fixes required, enabling a collar life of approximately 10 months. Our results are generally applicable to GPS‐based tracking studies of quadrupedal animals and could be applied to studies of energetics, behavioral ecology, and locomotion. This low‐cost approach overcomes the limitation of low fix rate GPS and enables the long‐term deployment of lightweight GPS collars.

## Introduction

Long‐term high‐resolution position data and accurate estimates of distance travelled are fundamental for understanding free ranging animal behavior, energetics, resource utilization, group dynamics, the nature of locomotor activity, and disease spread (Carbone et al. 2007; Hein et al. 2012). Tracking collars fitted to wild animals are required to operate for months or years to avoid the need for frequent battery (and hence collar) replacement, which is undesirable for animal welfare and financial reasons. As GPS modules are power‐hungry, the tight power budget restricts the number of GPS fixes that can be made and has forced long‐term studies to use low‐resolution position data (daily or hourly fix rates) (Nelson et al. 2004; Frair et al. 2005; Oksanen et al. 2015).

At low fix rates, linear interpolation (straight lines between consecutive fixes) cannot characterize the often tortuous path of an animal leading to underestimation of distance travelled, and hence average movement speed (Rowcliffe et al. 2012). For some species, this can result in distance travelled being underestimated by up to 93% (Rowcliffe et al. 2012).

Dead reckoning has been identified as a possible solution to this problem as it is a low power method which can provide high‐resolution position data (Frair et al. 2010; Rowcliffe et al. 2012). It uses measurements from micro‐electro‐mechanical systems (MEMS) accelerometers and magnetometers, which consume much less power than GPS modules, to estimate speed and heading. These estimates are combined to generate a velocity vector that can be integrated to give estimates of positions at frequent intervals. The accumulation of small errors in the speed and heading estimates causes the dead reckoned reconstructed path to suffer from drift error (Groves 2013). Infrequent GPS fixes (Constandache et al. 2010; Symington and Trigoni 2012) can be used to zero this drift.

Dead reckoning is a commonly used technique for estimating the path of aquatic animals (Wensveen et al. 2015) and for pedestrian navigation (Jiménez et al. 2009; Tian et al. 2014). However, we could only find one study that has applied dead reckoning with drift correction to quadrupeds (Bidder et al. 2015). This could be because of the difficulty in calculating speed from accelerometer measurements in quadrupeds. The step counting approach that works well with bipeds (Jiménez et al. 2009) does not translate to the diverse range of quadruped gaits. Using a speed proxy calculated from accelerometer measurements, Bidder et al. (2015) were able to calculate a dead reckoning solution for quadrupeds.

Previous work, however, has not explored the trade‐off between dead reckoning accuracy and power consumption. Without estimates of collar battery life, the suitability of the method for long‐term animal tracking studies cannot be determined.

The aim of the current study was to determine how accurately drift‐corrected dead reckoning can estimate high rate GPS position data. It also aimed to determine how much it can reduce bias in animal travel distance estimates, to measure power consumption, and to calculate realistic estimates of collar battery life.

We deployed animal tracking collars containing a GPS module and three‐axis accelerometers and magnetometers on freely exercising domestic dogs. Simulations were carried out to investigate how low fix rate GPS measurements affect path accuracy and estimates of distance travelled. Simulations were also used to measure the performance of drift‐corrected dead reckoning. Estimates of collar power consumption were made to show the suitability of this method for use in long‐term animal tracking studies.

## Methods

### Data collection

Nine medium‐sized domestic dogs (17.50–38 kg) were recruited for the study following an advertisement within the Royal Veterinary College (RVC). All dogs were walked regularly, able to exercise freely off the lead and had no locomotor or gait abnormalities. Measurements were made using an RVC GPS‐enabled wildlife tracking collar (Wilson et al. 2013) running modified firmware. The collar fitted around the neck of the dog and was adjusted to allow three fingers to fit between it and the animal's skin. The mass of the collar was 340 g. The collar contained a NEO‐M8N GPS module (u‐Blox, Thalwil, Switzerland) which provided position, velocity, and accuracy estimates at a rate of 5 Hz. It also contained a MMA8652FC three‐axis, 12 bit, digital accelerometer module (NXP Semiconductors, Eindhoven, Netherlands). This measured specific force with a ± 8 g range at a rate of 50 Hz. An HMC5883 three‐axis, 12 bit, digital magnetometer module (Honeywell, Plymouth, Minnesota, USA) measured magnetic field strength at a rate of 15 Hz. Sensors were controlled and data processed using a MSP430 16‐bit microcontroller (Texas Instruments, Dallas, Texas, USA), running custom software written in the “C” programming language. Data were saved to a memory card and then transferred to a personal computer for further processing. Data processing was carried out using MATLAB 2015a (Mathworks, Natick, Massachusetts, USA).

Each dog performed between three and eight trials of free exercise around familiar routes (different for each dog) on different days. A total of 2289 min of data were collected, ranging from 160 to 335 min per dog. GPS positions were converted from WGS84 latitude and longitude to local level coordinates, northings (*y*) and eastings (*x*), measured in meters. Poor quality (inaccurate) GPS data were identified by thresholding GPS module‐derived horizontal position accuracy estimates. GPS measurements with an accuracy estimate of >10 m standard deviation (SD) were removed. A total of 2261 min of data remained, ranging from 146 to 335 min per dog. During each trial, the dogs moved independently and choose their own speed, route, and gait. All dogs performed speeds indicative of walk, trot, and gallop (Maes et al. 2008) over mixed undulating terrain. The maximum speed we recorded was 17.84 m/s (lurcher) which is comparable to that found for racing greyhounds (18 m/s) (Usherwood and Wilson 2005) and for free ranging African wild dogs (19 m/s) (Hubel et al. 2016).

The RVC Clinical Research and Ethical Review Board approved the study (ref: 2013 1233).

### Collar power consumption

Power consumption was measured from the RVC collar board running a modified version of the wildlife collar firmware described in Wilson et al. (2013). The collar board was placed on a bench adjacent to a GPS repeater. Power consumption was determined by recording and averaging the voltage drop across a 0.2‐ohm resistor in the power line at the different GPS fix rates. While GPS power consumption is dependent on animal activity and satellite conditions, benchmarking revealed no consistent or significant operational differences between measurements made in the laboratory and on free ranging animals in the UK and Botswana. We used the same method when the collar was running the DCDR algorithm.

### Ground truth position data

The 5‐Hz GPS speed and position data were down‐sampled to 1 Hz, the 0.5 Hz anti‐aliasing filter removing unwanted high frequency components. While GPS data contain spatial errors, they are currently the best option to use as a benchmark measure of the position of free ranging animals. We assumed that these data (our ground truth) represented the animal's true path and that distanced travelled estimates would not contain bias (over‐ or underestimation error) (Ranacher et al. 2015).

### Low fix rate GPS data, position error, and proportional accuracy

We simulated low fix rate GPS data by down‐sampling the ground truth GPS data to rates of 720, 360, 240, 120, 60, 12, 6, 4, and 2 fixes per hour. The effect of using low fix rate GPS data on path accuracy was measured by calculating the 2‐dimensional root–mean‐squared (2D‐RMS) position error between the ground truth GPS path and the low fix rate GPS path. The 2D‐RMS error is calculated using

$$\text{2D‐RMS position error}=\sqrt{\frac{1}{N}\sum_{i=1}^{N}\left(\Delta E_{i}^{2}+\Delta N_{i}^{2}\right)}$$

where Δ*E*_*i*_ and Δ*N*_*i*_ are the errors in the east and north components of the *i* ^th^ position estimate fix and *N* is the number of fixes.

Proportional accuracy *D*_*p*_ was used to measure bias introduced into distance travelled estimates calculated from low fix rate GPS position data. The sum of all straight line distances between adjacent fixes represents an estimate of the true distance travelled (*d*_t_). The apparent distance travelled (*d*_a_) was calculated by taking the sum of all straight line distances between fixes for each of the lower fix rate GPS data. Proportional accuracy *D*_p_ is the ratio of the true distance travelled to the apparent distance traveled (Rowcliffe et al. 2012).

$$D_{p}=\frac{d_{a}}{d_{t}}$$

For this simulation *D*_p_ ranges between 0 and 1, with 1 being perfectly accurate.

### Dead reckoning

Dead reckoning (DR) combines tilt corrected heading (*φ*) estimates from the magnetometer with speed (*v*_DR_) estimates from the accelerometers to generate a velocity vector. This vector was generated at two‐second intervals as this provided a good compromise between accuracy and collar data storage. Note that heading was measured clockwise from north. The distances travelled in the east (*x*) and north (*y*) directions were calculated from the components of this vector (multiplied by the time interval to convert to distance) and added to the previous position (*x*_*i*_, *y*_*i*_) to estimate the new position (*x*_*i*+1_, *y*_*i*+1_). The process is given by:

$$x_{i+1}=x_{i}+\left(v_{\mathrm{DR}}\text{sin}\varphi \right)\Delta t$$

$$y_{i+1}=y_{i}+\left(v_{\mathrm{DR}}\text{cos}\varphi \right)\Delta t$$

### Speed estimation from accelerometer measurements

Measurements from the three‐axis accelerometer were combined by calculating the vector of the dynamic body acceleration (VeDBA) as this has been shown to have an approximately linear relationship with animal speed (Bidder et al. 2012; Qasem et al. 2012). The specific forces measured by the three accelerometers were segmented into 2 s windows (no overlap) (Bidder et al. 2012). The reaction to gravity was removed from each signal by subtracting the mean value of the data in the window (Gleiss et al. 2011). The remaining signal represents an estimate of the acceleration (cyclic with the animal's movement). The acceleration signals were combined by calculating VeDBA using

$$\text{VeDBA}=\sqrt{\left(A_{x}^{2}+A_{y}^{2}+A_{z}^{2}\right)}$$

where *A*_*x*_, *A*_*y*_ and *A*_*z*_ are the mean of the square of the acceleration values.

**Figure 1:**
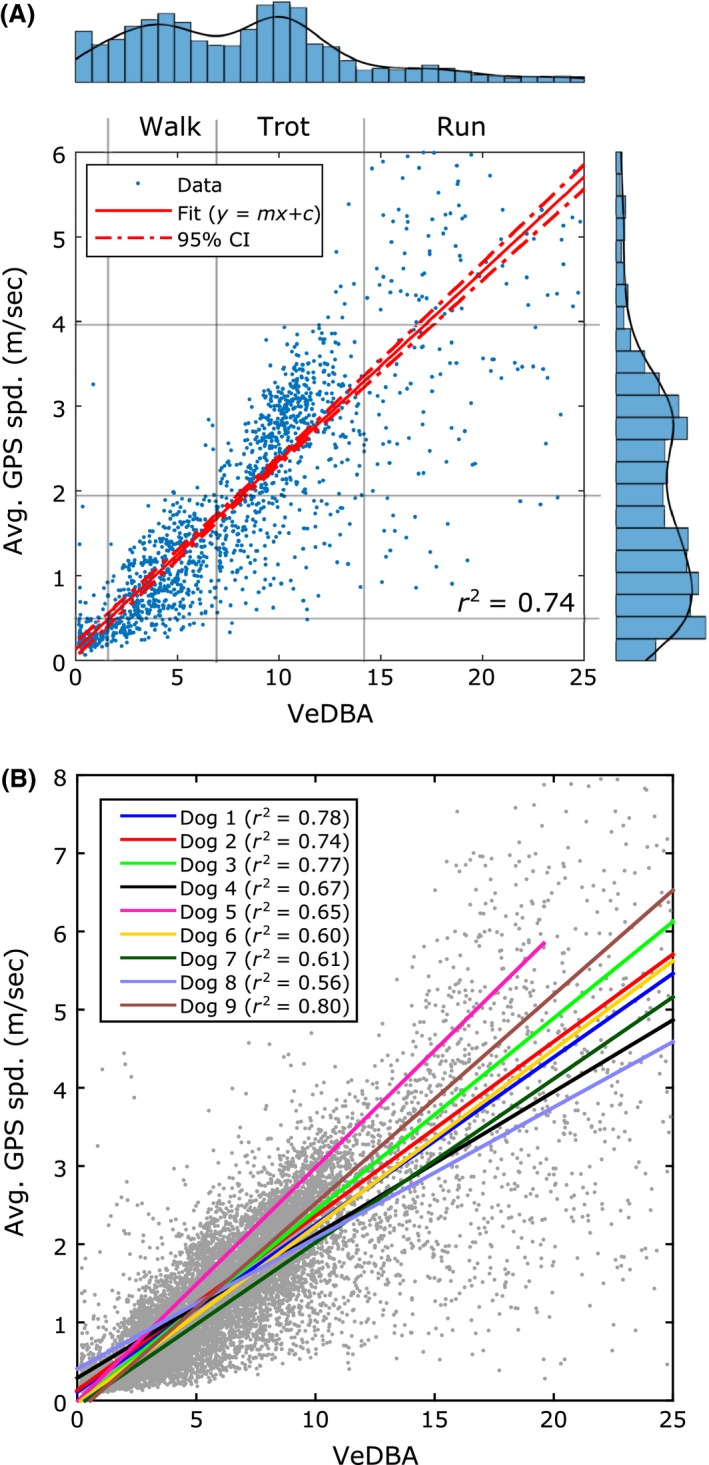
The relationship between GPS speed (mean over a 2‐s window) and the accelerometer speed proxy (VeDBA) for domestic dogs. (A) An example from dog 2. VeDBA was calculated over a 2‐s window. The linear fit to these data (training data) and 95% confidence intervals (CI) is represented by the solid red and dash dotted lines, respectively (*r* ^2 ^= 0.74). We use the distribution of the GPS speeds and VeDBA values (histograms with kernel density estimates marked by black lines) to determine areas on the scatter plot which represent walking, trotting, and running gaits. The local minimum of the kernel density estimate is taken as the transition point between walking and trotting. (B) Scatter plot showing the relationship between GPS speed and VeDBA for all dogs and the linear fit for each dog (D1 to D9).

The scatter plot in Figure 1A shows a representative example of the relationship between GPS speed (mean over a 2‐s window) and VeDBA (domestic dog 2) and a linear fit to the data (*r* ^2^ = 0.74). The distribution of the GPS speed and VeDBA values (histograms and kernel density estimates to the right and top of the scatter plot) is used as a guide to show which areas of the scatter plot represent walking, trotting, and running gaits (Maes et al. 2008). The local minimum of the kernel density estimate is used to represent the gait transition between walking and running (Fig. 1A). The bimodal distribution of the GPS speed measurements (Fig. 1A) suggests that domestic dog 2 is using preferred speeds (Hoyt and Taylor 1981) within its walking and trotting gaits. Similar results were found in the other dogs.

The gradient (*m*) and y‐intercept (*c*) of a linear model

$$y_{\mathrm{GPS}}=m\text{VeDBA}+c$$

were estimated using the least squares method, with *y*_GPS_ being the average GPS speed value in each 2 s window. The parameters of the model were estimated using the data from one randomly selected trail for each individual dog. Once the parameters of the linear model have been estimated, the model can be used to estimate speed (dead reckoning speed estimate, *y*_DR_). The relationship between GPS speed and VeDBA for the domestic dogs and the linear fits (training data) is shown in Figure 1B; the *r* ^2^ values range from 0.56 to 0.80. The accuracy of the dead reckoned speed estimate was measured in terms of residual error; this was calculated by subtracting the dead reckoned speed estimate from the GPS speed estimate.

### Heading estimation from magnetometer measurements

Magnetometers are subject to sensor bias, scale factor, and cross‐axis sensitivity errors, as well as hard and soft iron disturbances which distort the measurement of the earth's magnetic field; calibration is required to compensate for these factors (Ozyagcilar 2012).

Collar calibration took place prior to use and involved collection of 3 min of magnetometer data from the collar. During this time, the collar was rotated so that it moved through as many different orientations as possible relative to the earth's magnetic field. Collars were calibrated in an open space, away from potential external sources of magnetic disturbance. Full details of the calibration method can be found in the Appendix.

### Drift‐corrected dead reckoning

The accumulation of small errors in the speed and heading estimates causes the dead reckoning position estimate to drift with time (Groves 2013) (Fig. S1). Infrequent GPS position measurements (GPS fixes) can be used to zero this drift and also to improve the past dead reckoning position estimates (Constandache et al. 2010; Symington and Trigoni 2012). By concatenating all the data for all the trials completed by each dog, it was clear that the error in two‐dimensional position estimates accumulated in an approximately linear fashion over time (Fig. S1). Understanding that dead reckoning accuracy decreases with time and in a linear fashion, a linear drift correction algorithm (Constandache et al. 2010) was applied independently to the dead reckoning estimated path segments between GPS fixes.

## Results

**Figure 2:**
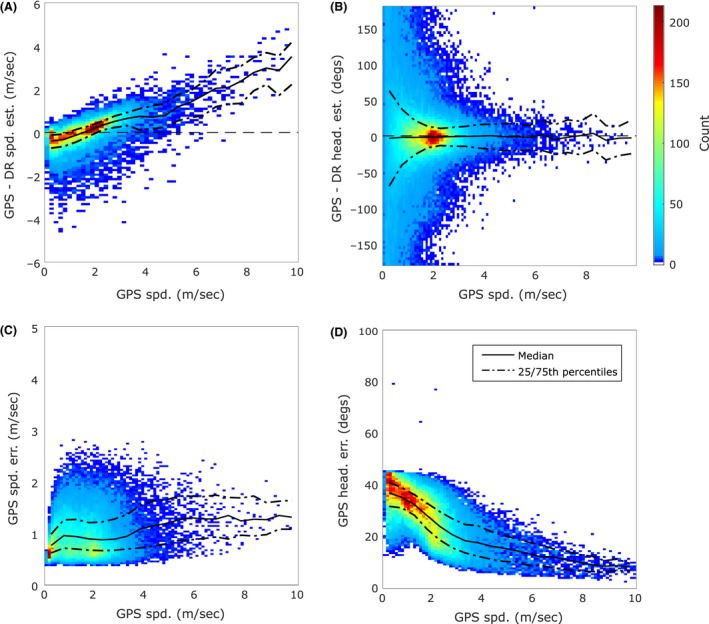
Dead reckoning accuracy and GPS module speed and heading accuracy estimates versus speed. Note that this figure shows the distribution of the residual signals from all nine domestic dogs. (A) The distribution of residual speed (GPS speed – Dead reckoning (DR) speed estimate). (B) The heading (GPS heading – DR heading estimate) errors. (C) The GPS module speed error estimate. (D) The GPS module heading accuracy estimates (note that the GPS module provides an absolute heading accuracy estimate). The GPS speed error estimate (C) shows only a slight increase in error with speed. This suggests that the trend in (A) is caused by errors in the DR speed estimate. DR tends to overestimate low speeds (0.4 to 1.6 m/s, walking gait) and underestimate medium to higher speeds (>1.6 m/s, trotting and running gaits) (A). The distribution of GPS module heading accuracy estimates shows more error at low speeds (D) and provides an explanation as to why the heading residual accuracy (B) is poor at low speeds (<2 m/s).

Figure 2 shows the distribution of the residual signals from all nine domestic dogs. The dead reckoned speed estimate tends to overestimate low movement speeds (0.4–1.6 m/s, walking gait) and underestimate medium to higher speeds (>1.6 m/s, trotting and running gaits). This trend can also be seen in Figure 1A. The bimodal GPS speed distribution shown in Figure 1A, suggesting preferred walking and trotting speeds, is also present in the distribution of the residual signals (Fig. 2A) from all nine domestic dogs. Figure 2C shows a slight increase in GPS module error with speed (median error of 1 m/s at 2 m/s to 1.5 m/s at >4 m/s). GPS speed module error was less than 0.9 m/s SD in 50% of the measurements (Fig. S2B, all dogs).

The heading residual, the difference between the GPS heading estimate and the dead reckoned heading estimate (provided by the magnetometer and tilt correction algorithm), is shown in Figure 2B. It should be noted that the GPS module actually provides an estimate of the animal's course and not its heading. However, in the current study, we take the animal's course and heading to be the same. GPS heading relies on movement and is inaccurate at low speed (Fig. 2D). Note that the GPS module provides an absolute heading accuracy estimate (Fig. 2D). The magnetometer should provide the most accurate heading estimate when it is stationary but this could not be confirmed by the current study because of the poor accuracy of the GPS heading estimates at low speeds (Fig. 2D). The dead reckoned heading estimate is most accurate (relative to GPS heading) when the dogs are trotting (Fig. 2B, between 1.6 and 3.5 m/s). It was assumed that the accuracy of the dead reckoned heading estimate would decrease substantially at higher speeds due to high dynamic movements corrupting the tilt correction algorithm. While there was a slight reduction in accuracy with higher speeds, it was not as great as had been expected (Fig. 2B). GPS module heading error was less than ± 29.65 degrees SD in 50% of the measurements (Fig. S2C).

**Figure 3:**
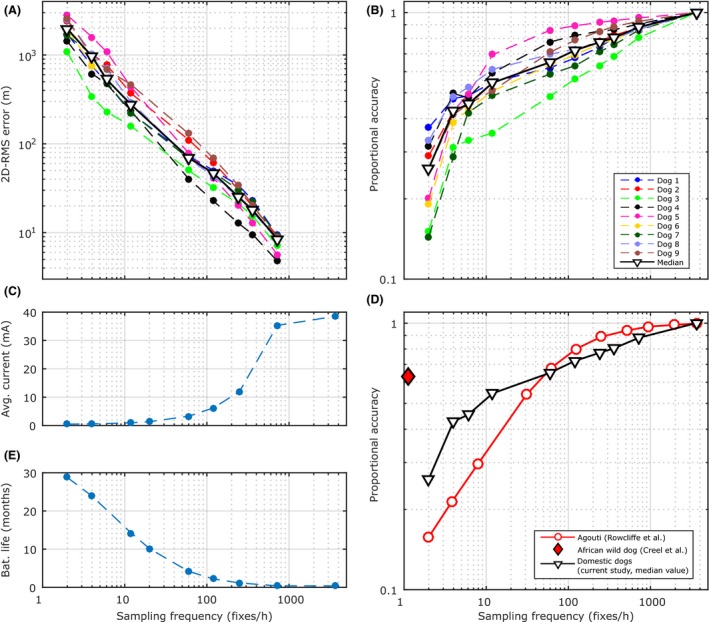
The effect of sampling frequency on 2D‐RMS position error, proportional accuracy, and collar battery life (GPS measurements only). (A) 2D‐RMS position error versus sampling fixes per hour. (B) Proportional accuracy for the domestic dogs versus sampling fixes per hour. Proportional accuracy (D_p_) is the ratio of the true distance traveled (d_t_) to the apparent distance travelled (d_a_) (Marcus Rowcliffe et al. 2012). Proportional accuracy varies between 0 and 1, with 1 being perfectly accurate. (C) Average collar current drawn. (D) Proportional accuracy for agouti (Marcus Rowcliffe et al. 2012), African wild dogs (Creel et al. 2013), and domestic dogs. (E) Collar battery life. Note the trade‐off between accurate position data and travel distance and collar battery life (A and E).

The result of simulations and measurements to show the effect that GPS sampling frequency has on 2D‐RMS position error, proportional accuracy, collar current consumption, and battery life is shown in Figure 3A,B,C,E. At low sampling frequencies, between 2 and 12 fixes per hour, 2D‐RMS position error and proportional accuracy are poor (median 2D‐RMS >275 m and median *D*_p_ < 0.55, Fig. 3A,B), but the low current requirement (Fig. 3C) results in a long battery life of between 28 and 14 months (Fig. 3E). More accurate estimation of path and distance travelled (median 2D‐RMS < 8.37 m and median *D*_p_ > 0.88, Fig. 3A,B) requires GPS fix frequencies of greater than 720 fixes per hour (1 fix every 5 sec), but high current consumption (Fig. 3C) reduces collar battery life to approximately 1 week (Fig. 3E).

Figure 3D shows how the proportional accuracy varies with sampling frequency for agouti, African wild dogs, and domestic dogs. The data points for the agouti were taken from the study by Marcus Rowcliffe et al. (2012), and the point for the African wild dog from the study by Creel et al. (2013). The proportional accuracy for the domestic dogs decreases more rapidly than for the agouti at high fix rates (between 3600 and 120 fixes per hour, Fig. 3D). It is similar at 60 fixes per hour (median proportional accuracy of 0.65 and 0.68 for domestic dogs and agouti, respectively, Fig. 3D). At fewer than 60 fixes per hour, the proportional accuracy of the domestic dogs decreases less rapidly than that estimated for the agouti (Fig. 3D).

**Figure 4:**
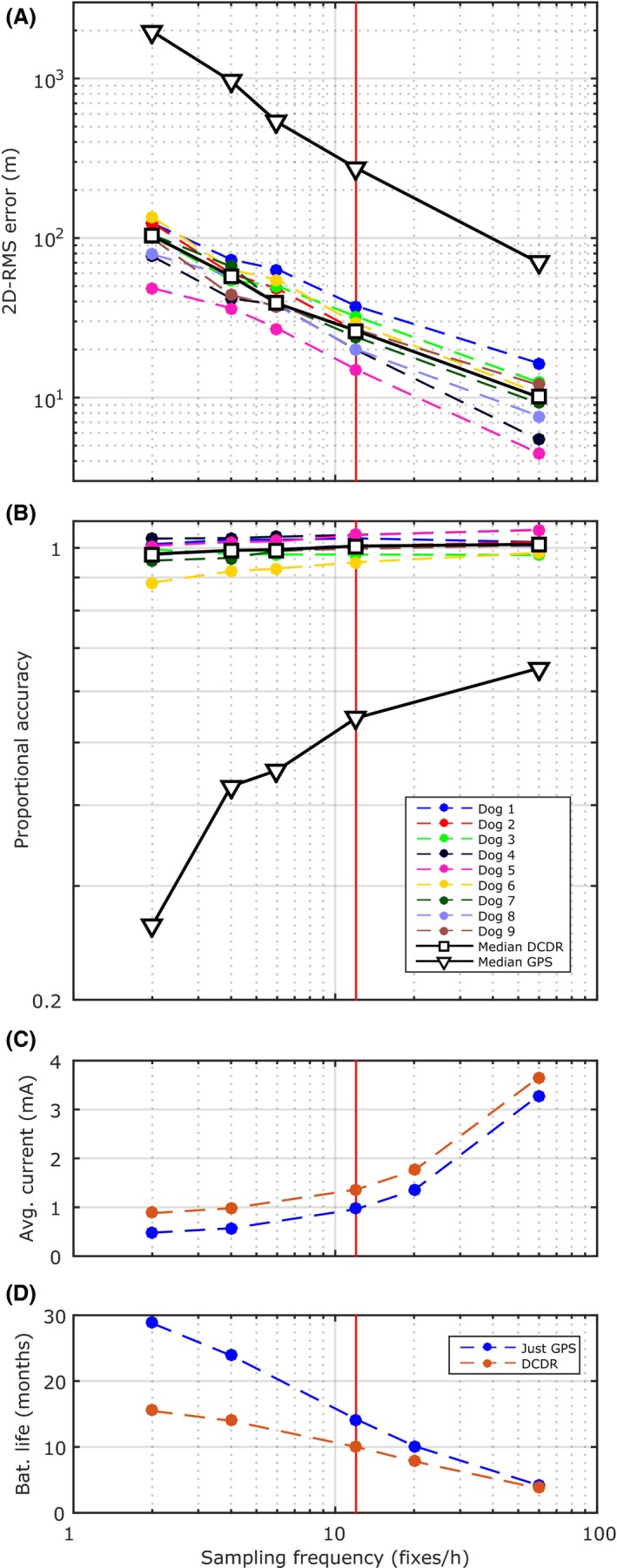
The effect of GPS and drift correction sampling frequency on 2D‐RMS position error, proportional accuracy, current consumption, and collar battery life (drift‐corrected dead reckoning). Note that while DCDR is a postprocessing method, it still requires extra code to be run on the microcontroller in the collar to generate, process, and save accelerometer and magnetometer data. This means that its current consumption will always be greater than that for just GPS data at a given sampling frequency. (A) Position accuracy of drift‐corrected dead reckoning (2D‐RMS error). (B) Proportional accuracy for the domestic dogs. Note the median values from “just GPS” data have been included in (A) and (B) to show the improvement in accuracy. (C) Collar current drawn for just GPS and drift‐corrected dead reckoning. (D) Collar battery life for just GPS and drift‐corrected dead reckoning.

Figure 4 shows how dead reckoning reduces 2D‐RMS position error and bias in estimates of distance while allowing the RVC collar to function for over a year. The drift‐corrected dead reckoning solution reduced 2D‐RMS position error and improved proportional accuracy for all domestic dogs (Fig. 4A,B). Proportional accuracy is relatively insensitive to drift correction sampling frequency (Fig. 4B). While drift‐corrected dead reckoning is a postprocessing method, it still requires extra code to be run on the microcontroller in the collar to generate, process, and save accelerometer and magnetometer data. This increases current consumption and reduces collar battery life (Fig. 4C and D, respectively) compared with just GPS measurements.

We have chosen to focus on the performance of the drift‐corrected dead reckoning method with a drift correction frequency of 12 fixes per hour (marked using vertical red line, Fig. 4). This provides more accurate fine‐scale path reconstruction (lower 2D‐RMS error, Fig. 4A), but at the cost of reducing RVC collar battery life to approximately 10 months. The improvement in performance, however, compared with low fix rate GPS (median GPS, Fig. 4A,B) is large (median 2D‐RMS position error of 26.30 m for DCDR compared with 274.96 m for GPS with a sampling frequency of 12 fixes per hour; a median proportional accuracy of 1.002 for DCDR compared with 0.55 for GPS with a sampling frequency of 12 fixes per hour). Individual domestic dog accuracy values for the drift‐corrected dead reckoning method with drift correction at 12 fixes per hour and low fix rate GPS (12 fixes per hour) are shown in Table 1.

**Table 1:** The accuracy of the drift‐corrected dead reckoning method (with a drift correction frequency of 12 fixes per hour) and low fix rate GPS (with a sampling frequency of 12 fixes per hour) for the individual domestic dogs

Dog	GPS (ground truth, 3600 fixes/hour)	DCDR (drift correction 12 fixes/hour)					Down‐sampled GPS (12 fixes/hour)				
	dist. (km)	2D‐RMS pos. error (m)	dist. (km)	diff. (km)	% diff.	prop. Acc.	2D‐RMS pos. error (m)	dist. (km)	diff. (km)	% diff.	prop. Acc.
	dt		da	dt‐da		Dp		da	dt‐da		Dp
1	16.15	37.51	16.77	−0.62	−3.83	1.04	220.23	8.76	7.40	45.78	0.54
2	21.13	26.30	21.17	−0.04	−0.20	1.00	369.43	11.55	9.58	45.32	0.55
3	10.67	32.29	10.43	0.25	2.32	0.98	158.07	3.78	6.89	64.54	0.35
4	11.87	19.94	12.41	−0.54	−4.54	1.05	233.48	7.08	4.79	40.35	0.60
5	19.52	15.01	20.42	−0.90	−4.61	1.05	433.99	13.59	5.94	30.42	0.70
6	13.49	29.14	12.86	0.62	4.61	0.95	274.96	8.36	5.12	37.98	0.62
7	16.42	23.99	16.34	0.08	0.51	0.99	222.82	8.00	8.43	51.30	0.49
8	18.68	20.09	18.84	−0.16	−0.85	1.01	293.89	11.41	7.26	38.90	0.61
9	21.36	26.82	21.35	0.01	0.03	1.00	463.25	10.89	10.47	49.03	0.51
25^th^ percentile	13.08	20.05	12.75	−0.56	−4.01	0.99	222.17	7.77	5.73	38.67	0.50
median	16.42	26.30	16.77	−0.04	−0.20	1.00	274.96	8.76	7.26	45.32	0.55
75^th^ percentile	19.92	29.93	20.61	0.12	0.96	1.04	385.57	11.45	8.71	49.60	0.61

RMS, root‐mean‐squared.

**Figure 5:**
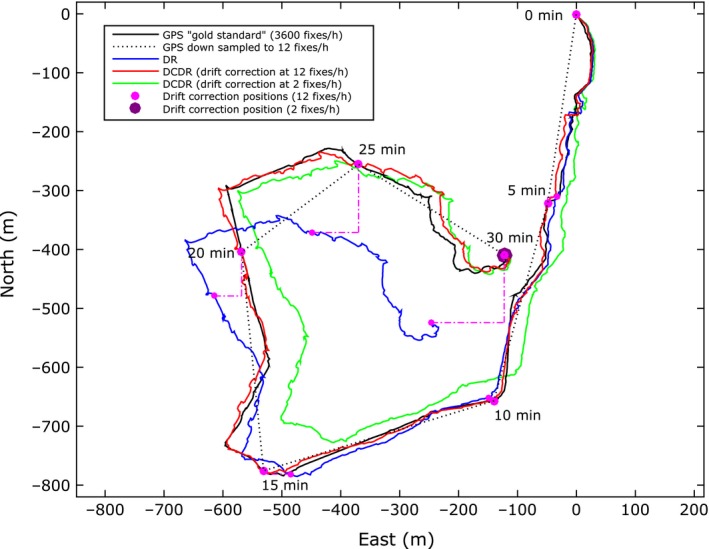
An example of gold standard and down‐sampled GPS paths, dead reckoned (DR), and two drift‐corrected dead reckoned (DCDR) paths from a domestic dog. This example is taken from domestic dog 5, trial 2. The gold standard GPS path has a sampling frequency of 3600 fixes per hour (1 Hz) and a true distance travelled (d_t_) of 2.43 km. The down‐sampled GPS path shows the problem of using low sample rate GPS data for distance estimation. It was created by down‐sampling the gold standard GPS path to 12 fixes per hour. The apparent distance travelled (d_a_) calculated from this path is 1.99 km. The DR path drifts away from the gold standard GPS path as errors accumulate with time. This drift is reduced by applying a linear correction to the DR path between GPS position fixes (DCDR paths). The drift correction points are marked using magenta circles. Using points taken at 2 and 12 fixes per hour makes little difference to the apparent distances travelled (2.43 and 2.57 km, respectively), and hence, the proportional accuracy values are similar (0.99 and 1.05, respectively). Using drift correction with the higher GPS position fix rate, however, improves the ability of the dead reckoned path to follow the gold standard GPS path. For this example, DCDR with drift correction at 2 fixes per hour has a 2D‐RMS position error of 54.3 m; with 12 fixes per hour position error is reduced to only 12.9 m.

Figure 5 shows an example of a ground truth and a down‐sampled GPS path, dead reckoned, and two drift‐corrected dead reckoned paths (with drift correction at 2 and 12 fixes per hour). This example is taken from dog 5, trial 2. The ground truth GPS path has a sampling frequency of 3600 fixes per hour (1 Hz). The true distance travelled (d_t_) calculated from this path is 2.43 km. The down‐sampled GPS path is shown to illustrate the problem of using low fix frequency GPS data for distance estimation (Fig. 5). It was created by down‐sampling the ground truth GPS path to a rate of 12 fixes per hour. The apparent distance travelled (d_a_) calculated from this path is 1.99 km, an underestimation of 0.44 km (a proportional accuracy of 0.82). The dead reckoned path drifts away from the ground truth GPS path as errors in the speed and heading estimates accumulate with time (Fig. 5). This drift is reduced by applying a linear correction between GPS drift correction fixes taken at regular intervals. The results of drift correction at intervals of 2 and 12 fixes per hour are shown in Figure 5. Drift correction points are marked using magenta circles. The apparent distance travelled for the drift‐corrected dead reckoned paths with drift correction at 2 and 12 fixes per hour is 2.43 and 2.57 km, respectively (proportional accuracy of 0.99 and 1.05, respectively). Using the higher drift correction sample rate of 12 fixes per hour improves the reconstruction to more accurately match the ground truth GPS path (Fig. 5) (2D‐RMS position error of 54.33 and 12.94 m at 2 and 12 fixes per hour).

## Discussion

Accurate measurement of animal position and distance travelled requires very frequent location fixes (many fixes per minute) (Rowcliffe et al. 2012). In long‐term animal tracking studies that use collar‐mounted GPS modules, there is a trade‐off between fix rate and power consumption, and hence battery size and study duration. As GPS is power‐hungry, previous studies (Nelson et al. 2004; Frair et al. 2005; Oksanen et al. 2015) have used low fix rates of between 1 and 4 measurements per hour to conserve battery life and allow long‐term data collection. We have shown that, for the domestic dogs in our study, with 2 fixes per hour, 2D‐RMS position error is 1970 m (median) and distance travelled is underestimated by 74% (median) (82/68%; 25/75 percentiles). This is caused by the inability of linear interpolation to represent the often tortuous path of the animal. Furthermore, at such low sample rates, data relating animal position to features on the ground may contain large errors and could therefore be misleading.

Comparison of our results with those from other studies (Rowcliffe et al. 2012; Creel et al. 2013) all show the same trend – that calculating distance travelled from low sample rate data results in biased measurements (Fig. 3B,D). Differences can be explained as the studies used different measurement techniques (camera trap and model, GPS measurements, and GPS/odometer), species, habitat types, and movement patterns.

The current study has shown that drift‐corrected dead reckoning provides a promising solution to the problem of distance underestimation. We have extended previous work (Bidder et al. 2015) by exploring the trade‐off between dead reckoning accuracy and power consumption and shown the suitability of the method for long‐term studies of wild quadrupeds. The method uses low power (and cost) accelerometers and magnetometers to estimate speed and heading at high sample rates (every 2 s). Path drift is zeroed, and position estimates are improved by applying a linear drift correction method between low fix rate GPS position measurements. Reducing GPS fix rate allows collar battery life to be extended. We have shown that drift‐corrected dead reckoning with a GPS drift correction update every 5 min provides high temporal resolution and accurate estimation of the animal's path with a median 2D‐RMS position error of 26.30 m over a median path length of 16.43 km. Such measurements provide a basis for determining time, location, and duration of contact between animals which are important for furthering our understanding of the process of disease spread, group social structure, and interactions and habitat utilization.

We have also shown that drift‐corrected dead reckoning with a GPS drift correction update every 5 min provides a median proportional accuracy of 1.002. This means that distance travelled is only overestimated by 0.2% (median). It should be noted that collar life (for the collars in Wilson et al. 2013) using this method would be 10 months. It should be possible to extend this time to over a year by reducing the GPS drift correction fix rate when the animal is stationary (sleeping or resting). This could be carried out using a simple behavior/locomotion detection algorithm on the signals measured by the accelerometers as detailed in Wilson et al. (2013). To achieve a similar level of accuracy using GPS measurements alone would require 12 fixes per minute and result in a battery life of approximately 11 days.

Measurement error in individual position fixes could cause the overestimation of distance traveled (on average) (Ranacher et al. 2015). Its effect on our estimate of the true distance travelled was investigated by comparing distance estimates from GPS position data, Kalman smoothed (KS) position data (fusion of 5 Hz GPS data with 250 Hz accelerometer and gyroscope data), and from GPS velocity measurements on an example representative trial (trial 1, dog 1). KS position data and GPS velocity measurements provide the best available benchmarks. The Kalman smoother (Wilson et al. 2013) reduced position error estimates from less than 3.1 m SD to less than 0.4 m SD in 50% of the measurements (Fig. S1A). GPS speed measurements will not suffer from the same overestimation effect (Ranacher et al. 2015). Distance travelled estimates calculated by integrating the speed measurements are potentially more accurate than those from position data (Ranacher et al. 2015). The distance travelled estimates from the GPS position data, Kalman smoothed position data, and the integrated GPS velocity data were 5.905 km, 5.731 km, and 5.819 km, respectively (representative trial). As the difference between these values is small (a maximum difference of 174 m between GPS and Kalman distance), we believe that the ground truth position data provide a reasonably accurate estimate of the true distance traveled.

Generalization of the drift correction dead reckoning method to other animals depends on the correlation between the animal's speed and the speed proxy (VeDBA) derived from the accelerometer measurements. This should be successful as previous work has shown that such a correlation exists in a wide range of quadrupeds and bipeds (Bidder et al. 2012; Qasem et al. 2012). A similar method was found to work successfully on domestic dogs, badgers, and horses (Bidder et al. 2015). The advantage of the DCDR method compared to some other underestimation solutions (Pépin et al. 2004; Rowcliffe et al. 2012) is that the speed and heading estimates are continually being updated (in our study every 5 min) by the GPS drift correction position. Changes in habitat or behavior should therefore have little effect on distance or path estimates.

Other approaches could be used to replace the low rate GPS position fixes used for drift correction. An array of fixed RFID readers would deliver time‐stamped locations and enable a tag that requires much lower power and is much smaller in physical size than a GPS‐based tag. This opens up the potential for studies of disease transmission and social structure and hierarchy in animals as small as mice, rats, and many birds. Likewise, photographic approaches such as camera or video traps could provide equivalent occasional location data for drift correction. This would enable studies of small animals and those in GPS‐deprived areas to be carried out. Reducing reliance on GPS has advantages for power management and tag size and removes the problem of the non‐Gaussian nature of GPS noise. At the smaller scale, the higher spatial accuracy of a few centimeters provided by photographic techniques (Rowcliffe et al. 2012) may be required for accurate path reconstruction. For animals that spend time in groups and have regular close proximity encounters, encounter‐based drift correction fixes could be used (Symington and Trigoni 2012). This approach could be implemented by adding short‐range radio link transmitters/receivers to the collars (Prange et al. 2000). Such radio techniques could provide range through time of flight or signal strength between a collar and other collars or fixed points. All these approaches would reduce the issue of intermittent GPS coverage caused by canopy closure and would significantly reduce power consumption.

It may be possible to improve the performance of the drift correction algorithm by identifying behavior or gait of the animal associated with lower accuracy dead reckoning and increasing the GPS fix rate to correct this. Using a variable drift correction, rate that is optimized according to behavior or gait could also contribute to collar power management strategies. Data fusion techniques, such as variants of the Kalman filtering applied in the current study, also have the potential to improve the accuracy of the system.

## Conclusion

Drift‐corrected dead reckoning is a low power approach that overcomes the limitations of low fix rate GPS. It substantially improves the accuracy of measurement of animal position and distance travelled and enables the long‐term deployment of lightweight GPS tracking collars.

## Conflict of Interest

None declared.

## Supporting information


**Figure S1**. Propagation of 2D horizontal error with time.

**Figure S2**. GPS accuracy estimates from data collected from free ranging domestic dogs. Empirical cumulative distribution functions (CDFs) are used to show the distribution of the standard deviation (SD) error estimates provided by the GPS module (marked GPS in the legend) and the Kalman smoothing (KS) algorithm. (A–C) show position, speed and heading error estimates respectively. The Kalman smoother reduces position (A) and speed (B) error by fusing GPS, accelerometer and gyroscope data. This improvement is shown using an example data set (dog 1, trial 1).

## Detailed methods

### Magnetometer calibration and tilt correction

In an ideally calibrated sensor, these data would resemble a sphere centerd on zero with a radius equal to the local magnetic field strength. Fitting an ellipsoid to these data gave the vector to the center of the ellipsoid (bias error ***B***), unit vectors of the three principal axes (${\hat{e}}_{x}$, ${\hat{e}}_{y}$ and ${\hat{e}}_{z}$) and the radii ($r_{x}$, $r_{y}$ and $r_{z}$) of those principal axes. Combining the unit vectors as the columns in a direction cosine matrix (DCM), ***E***_DCM_, the calibration parameter ***M*** was calculated:

$$M=E_{\mathrm{DCM}}\left[\begin{array}{ccc} \frac{1}{r_{x}} & 0 & 0\\ 0 & \frac{1}{r_{y}} & 0\\ 0 & 0 & \frac{1}{r_{z}} \end{array}\right]{E_{\mathrm{DCM}}}^{T}$$

Each vector of calibrated magnetometer data (***H***_calib_) was then calculated from raw measured data (***H***_raw_) as:

$$H_{\mathrm{calib}}=M\left(H_{\mathrm{raw}}-B\right)$$

This, when applied to the raw calibration data, centers the data on the origin and scales the data along the principal axes to resemble a unit sphere.Magnetometer measurements give the direction of a magnetic field in the sensor axes. Heading is defined as the angle (right‐handed about the vertical down axis) between north and the sensor x axis projected onto the horizontal plane. Mean calibrated accelerometer measurement vectors ($\overset{¯}{A}$) were calculated over 2‐s intervals and assumed to give the reaction to gravity that also defines the horizontal plane. From the point of view of the tilted sensor frame, the vertical down axis direction (***z***_down_) was:

$$z_{\mathrm{down}}=-\overset{¯}{A}$$

Any vector on the sensor x axis was coplanar with ***z***_down_ and the level x axis in the sensor frame (***x***_level_). Taking the cross‐product of ***z***_down_ and a dummy x axis vector gave the level y axis (***y***_level_), which in turn gave the level x axis:

$$y_{\mathrm{level}}=z_{\mathrm{down}}\times \left[\begin{array}{c} 1\\ 0\\ 0 \end{array}\right]$$

$$x_{\mathrm{level}}=y_{\mathrm{level}}\times z_{\mathrm{down}}$$

Assuming that accelerometer and magnetometer axes are aligned, a DCM (***T***_DCM_) was constructed from unit vectors to transform from sensor axes to the level frame:

$$T_{\mathrm{DCM}}={\left[\begin{array}{ccc} {\hat{x}}_{\mathrm{level}} & {\hat{y}}_{\mathrm{level}} & {\hat{z}}_{\mathrm{down}} \end{array}\right]}^{T}$$

A mean calibrated magnetometer measurement ($\overset{¯}{H}$) was also calculated over 2‐s intervals. This was transformed to give a vector equivalent to that which would have been measured with the magnetometer X and Y axes leveled in the horizontal plane (${\overset{¯}{H}}_{\mathrm{horiz}}$:

$${\overset{¯}{H}}_{\mathrm{horiz}}=T_{\mathrm{DCM}}\overset{¯}{H}$$

Heading (*φ*) was then calculated using a four quadrant inverse tangent to determine the angle of the x axis to the horizontal component of the magnetic field in a 0 to 2*π* range:

$$\varphi =\left\{\begin{array}{cc} -{\text{tan}}^{-1}\left(\frac{{\overset{¯}{H}}_{{\mathrm{horiz}}_{y}}}{{\overset{¯}{H}}_{{\mathrm{horiz}}_{x}}}\right) & \text{if}\;{\overset{¯}{H}}_{{\mathrm{horiz}}_{x}}\geq 0\;\;\text{and}\;\;{\overset{¯}{H}}_{{\mathrm{horiz}}_{y}}\leq 0,\\ \pi -{\text{tan}}^{-1}\left(\frac{{\overset{¯}{H}}_{{\mathrm{horiz}}_{y}}}{{\overset{¯}{H}}_{{\mathrm{horiz}}_{x}}}\right) & \text{if}\;{\overset{¯}{H}}_{{\mathrm{horiz}}_{x}}<0,\\ 2\pi -{\text{tan}}^{-1}\left(\frac{{\overset{¯}{H}}_{{\mathrm{horiz}}_{y}}}{{\overset{¯}{H}}_{{\mathrm{horiz}}_{x}}}\right) & \text{if}\;{\overset{¯}{H}}_{{\mathrm{horiz}}_{x}}\geq 0\;\text{and}\;\;{\overset{¯}{H}}_{{\mathrm{horiz}}_{y}}>0 \end{array}\right.$$

### Linear drift‐corrected dead reckoning

Following the derivation in Symington and Trigoni (2012), let $\tilde{P}\left(t\right)$be the current dead reckoned path segment. Let *t*_*i*_ and *t*_*i*+1_ be the time of two GPS fixes, with coordinates *X*(*t*_*i*_) and *X*(*t*_*i*+1_). Linear drift correction shifts the dead reckoned path segment so that it starts at the first GPS fix, *X*(*t*_*i*_). It then calculates the difference between the GPS, *X*(*t*_*i*+1_), and dead reckoned end points and adds a proportion of this (linearly in time) to the dead reckoned path segment, $\tilde{P}\left(t\right)$. This is achieved using the shift vector $\vec{v}=X\left(t_{i}\right)-\tilde{P}\left(t\right)$ and the correction vector $\vec{c}=X\left(t_{i+1}\right)+\vec{v}-\tilde{P}\left(t_{i+1}\right)$. The corrected path is thus given by

$$P\left(t\right)=\tilde{P}\left(t\right)+\vec{v}+\frac{t-t_{i}}{t_{i+1}-t_{i}}\vec{c}\;t_{i}\leq t\leq t_{i+1}$$
